# Non-digestible oligosaccharides directly regulate host kinome to modulate host inflammatory responses without alterations in the gut microbiota

**DOI:** 10.1186/s40168-017-0357-4

**Published:** 2017-10-10

**Authors:** Richard Y. Wu, Pekka Määttänen, Scott Napper, Erin Scruten, Bo Li, Yuhki Koike, Kathene C. Johnson-Henry, Agostino Pierro, Laura Rossi, Steven R. Botts, Michael G. Surette, Philip M. Sherman

**Affiliations:** 1 Cell Biology Program, Research Institute, Division of Gastroenterology, Hepatology and Nutrition, Hospital for Sick Children, 555 University Avenue, Toronto, ON M5G 1X8 Canada; 2 Department of Laboratory Medicine and Pathobiology, Faculty of Medicine, University of Toronto, Toronto, Canada; 3 Physiology and Experimental Medicine, Research Institute, Hospital for Sick Children, Toronto, ON Canada; 4 Vaccine and Infectious Disease Organization-International Vaccine Center, University of Saskatchewan, Saskatoon, SK Canada; 5 Department of Biochemistry, University of Saskatchewan, Saskatoon, SK Canada; 6 Division of General and Thoracic Surgery, Hospital for Sick Children, Toronto, ON Canada; 7 Faculty of Dentistry, University of Toronto, Toronto, ON Canada; 8 Biology Department, Burman University, Lacombe, AB Canada; 9 Department of Medicine, Faculty of Health Sciences, McMaster University, Hamilton, ON Canada; 10 Department of Biochemistry and Biomedical Sciences, Faculty of Health Sciences, McMaster University, Hamilton, ON Canada

**Keywords:** Prebiotics, Kinome, Non-digestible oligosaccharides, *E. coli*, Lipopolysaccharide

## Abstract

**Background:**

Prebiotics are non-digestible food ingredients that enhance the growth of certain microbes within the gut microbiota. Prebiotic consumption generates immune-modulatory effects that are traditionally thought to reflect microbial interactions within the gut. However, recent evidence suggests they may also impart direct microbe-independent effects on the host, though the mechanisms of which are currently unclear.

**Methods:**

Kinome arrays were used to profile the host intestinal signaling responses to prebiotic exposures in the absence of microbes. Identified pathways were functionally validated in Caco-2Bbe1 intestinal cell line and in vivo model of murine endotoxemia.

**Results:**

We found that prebiotics directly regulate host mucosal signaling to alter response to bacterial infection. Intestinal epithelial cells (IECs) exposed to prebiotics are hyporesponsive to pathogen-induced mitogen-activated protein kinase (MAPK) and nuclear factor kappa B (NF-κB) activations, and have a kinome profile distinct from non-treated cells pertaining to multiple innate immune signaling pathways. Consistent with this finding, mice orally gavaged with prebiotics showed dampened inflammatory response to lipopolysaccharide (LPS) without alterations in the gut microbiota.

**Conclusions:**

These findings provide molecular mechanisms of direct host-prebiotic interactions to support prebiotics as potent modulators of host inflammation.

**Electronic supplementary material:**

The online version of this article (10.1186/s40168-017-0357-4) contains supplementary material, which is available to authorized users.

## Background

The concept of altering the gut microbiota to improve human health has led to a spectrum of therapeutic strategies, one of which includes the consumption of plant-derived prebiotics [1]. Prebiotics are oligosaccharides that resist intestinal digestion and absorption to reach the colon virtually intact to act as growth substrates for certain health-promoting bacterial communities [2]. Prebiotic consumption, particularly in early life, produces beneficial effects on the development of microbiota and immune function, but the mechanisms are ill-defined [3]. It is traditionally believed that the two effects are intertwined, where immune modulation is an indirect result of the changes in microbiota, while little is known about the possibility of direct interactions with the host.

The first indication of direct host-prebiotic interactions came from the finding that oligosaccharides derived from milk directly induce cord blood T cell maturation and cytokine production [4]. Since then, studies have shown that in addition to T cells, prebiotic exposure also triggers cytokine production in dendritic cells (DCs), IECs and monocytes [5–7]. More recently, several animal studies have also shown that non-digestible oligosaccharides can regulate B cell responses and macrophage markers even in germ-free animals devoid of microbes [8, 9], suggesting that prebiotics could have direct immune-modulatory effects beyond their microbial properties, though the mechanisms of which are currently unknown.

Recently, we demonstrated that prebiotic exposures can directly stimulate host protein kinase C phosphorylation to mediate changes on barrier function [10], suggesting a connection between kinase activities (the kinome) and the host response to prebiotics. However, it is unclear to what extent the host kinases are modulated by prebiotics and how this may relate to host immunity. Kinases phosphorylate a wide selection of substrates to alter protein function and can rapidly influence a diverse repertoire of biological processes and cellular signaling [11]. To thoroughly investigate these processes, we employed a systems biology approach to address the outstanding questions of host-prebiotic interactions.

In this study, we explored prebiotic signaling by performing kinome analysis on IECs in response to two commercial prebiotic oligosaccharides of varying length: inulin and short-chain fructooligosaccharide (scFOS), with and without challenge by the enteric pathogen enterohemorrhagic *Escherichia coli* serotype O157:H7 (EHEC). This was done using a customized peptide microarray containing 282 unique phosphorylation substrates (each peptide replicated nine times) to profile host kinome activities [12]. Through this analysis, we identified specific kinases (nodes) whose phosphorylation levels were directly modulated by prebiotic exposure in a glycan-specific manner. The functional annotations of these nodes revealed several relevant networks and pathways, and these predicted pathways were further validated both in vitro using Caco-2Bbe1 cell line and in vivo using endotoxemic mice [13]. Together, our findings demonstrate that prebiotics directly impact host signaling to affect mucosal inflammation, which provides a molecular explanation for how prebiotic consumption regulates host inflammation independent of gut microbiota.

## Results

### Prebiotics modulate host kinome in a glycan-specific manner

**Fig. 1:**
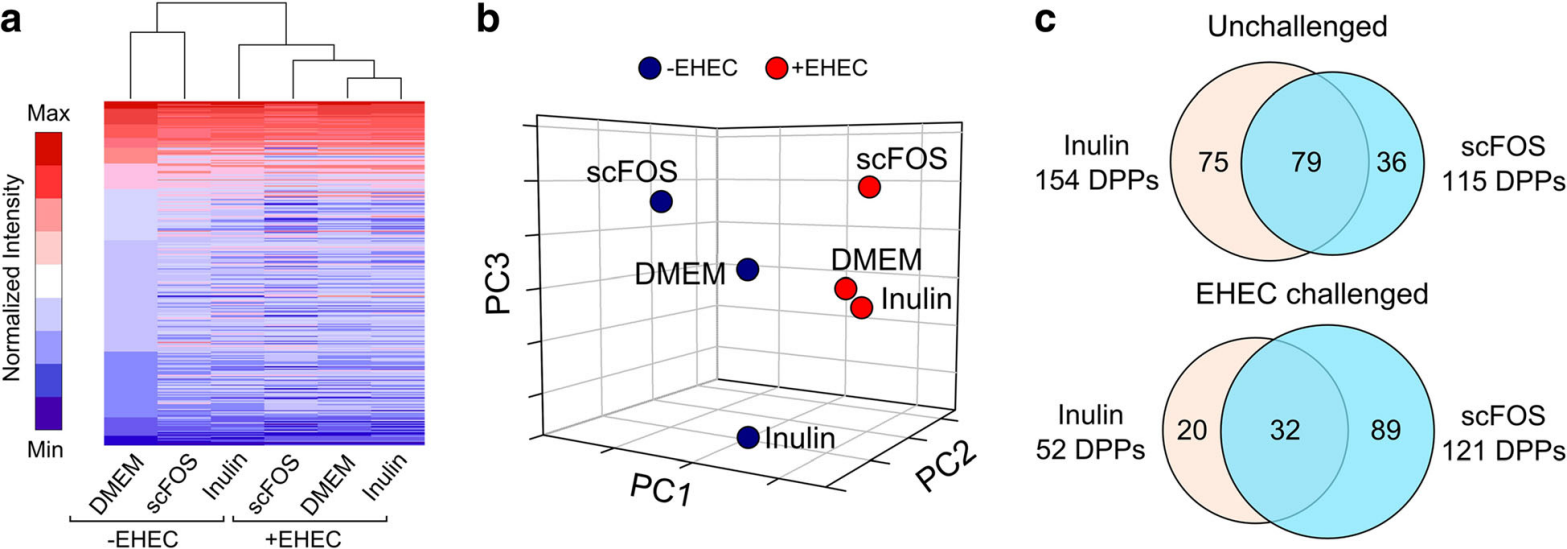
Kinome response of prebiotic-treated IECs to EHEC O157:H7 challenge. **a** Heatmap view displaying the host kinome activities of Caco-2Bbe1 cells in response to prebiotic pre-exposure and EHEC infection (MOI of 10:1, 3 h) (*n* = 3). **b** Principle coordinates analysis displaying the separation of host signaling by inulin and scFOS within both unchallenged and EHEC-challenged cells. **c** Venn diagram illustrating the breakdown of DPPs identified from the initial kinome screening

To measure host kinome response to prebiotics, we cultured Caco-2Bbe1 cells in cell media with or without inulin or scFOS (16 h, 100 mg ml^−1^), and challenged cells with EHEC (MOI of 10:1) for 3 h—a time-point previously shown to trigger host signaling responses [14]. In general, inulin and scFOS-treated cells exhibited distinct kinome profiles (Fig. 1a). This was particularly evident after the EHEC challenge whereby EHEC control cells clustered closely with inulin but not with scFOS (Fig. 1b), indicating a difference between the two prebiotics in altering the host signaling response to the EHEC challenge. To further investigate this difference, we compared the statistically significant peptides, otherwise referred to as differentially phosphorylated peptides (DPPs). In total, we found 233 DPPs in prebiotic-treated cells that had significant fold changes relative to either unchallenged or EHEC-challenged cells (Additional file 1: Figure S1A). Although a majority of DPPs (79 DPPs) were common between inulin and scFOS in the unchallenged state (Fig. 1c), only 32 DPPs were shared during the EHEC challenge with 89 DPPs being unique to scFOS.

To compare the DPPs between inulin and scFOS, we constructed volcano plots and identified the three most up- and down-phosphorylated kinases (Additional file 1: Figure S1B-E). In the unchallenged state, inulin and scFOS both decreased the phosphorylation of JUN, a central mediator in inflammation control, and increased phosphorylation of interferon regulatory factor 3 (IRF-3), a transcription factor that regulates interferon-gamma (IFN-γ)-related genes during inflammation. But aside from these two commonalities, the most extreme DPPs between inulin and scFOS exposures were vastly different. For example, the most down-phosphorylated peptide during inulin exposure was receptor for activated C kinase (RACK1), as opposed to interleukin-1 receptor-associated kinase 1 (IRAK4) for scFOS. Taken together, these results suggest that prebiotic directly elicits widespread changes to the host kinome in a manner that is unique to the prebiotic structure.

### Prebiotic modulation of the kinome alters host cell functions

**Fig. 2:**
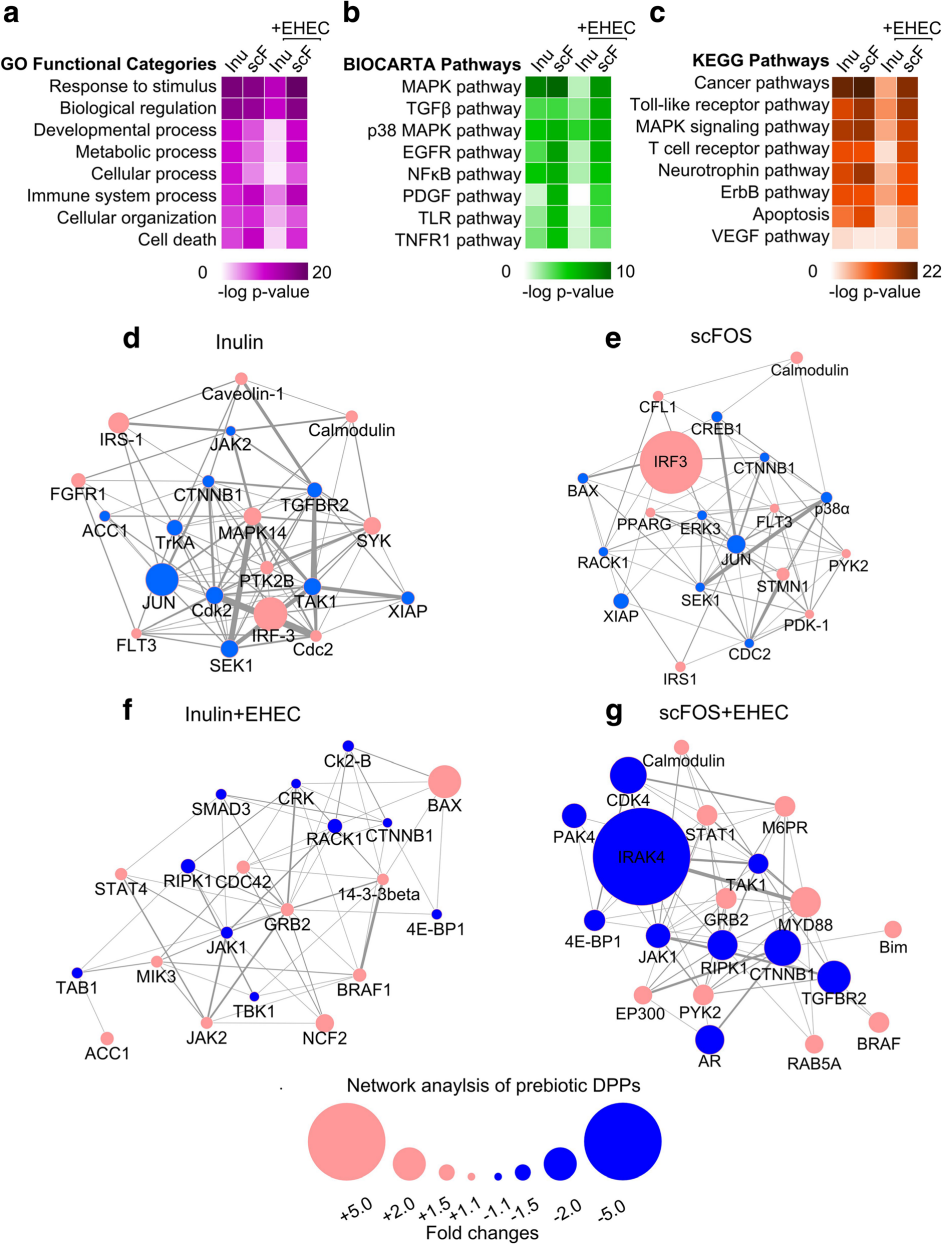
Biological functions of prebiotic-induced kinome responses. **a** Gene ontology annotations using DAVID of the functional categories modulated by inulin or scFOS as shown in a heatmap view. **b**, **c** Pathways most enriched by the kinases modulated by inulin or scFOS were annotated using Biocarta and KEGG databases and shown as a heatmap view. **d**–**g** Protein network displaying the 20 kinases most modified by inulin or scFOS in both unchallenged and EHEC-challenged states

To predict the biological consequence of the prebiotic changes to host kinome, we functionally annotated the Gene Ontology (GO) categories and affected pathways using DAVID [15]. While there were surprising overlaps in functions and pathways in the unchallenged state, we noted a drastic divergence between inulin and scFOS-mediated host functions during the EHEC challenge. For example, the GO categories most affected by inulin were limited to *response to stimulus* and *biological regulation*, whereas in scFOS-treated cells, additional processes such as *metabolic process*, *cellular process*, and *immune system process* were highly enriched (Fig. 2a). Similarly, when we queried the affected pathways, we found that scFOS was predicted to strongly affect a greater number of pathways including the mitogen-activated protein kinase (MAPK) pathway, Toll-like receptor (TLR), nuclear factor kappa B (NF-κB) pathway, transforming growth factor beta (TGFβ), and the epidermal growth factor receptor (EGFR), whereas in inulin-treated cells, only the p38 MAPK pathway was enriched (Fig. 2b). To confirm this difference, we also queried the identified DPPs alternatively using the Kyoto Encyclopedia of Genes and Genomes (KEGG) database, and in agreement with the Biocarta results, only MAPK signaling was enriched in the inulin treatment (Fig. 2c).

To further differentiate the signaling responses between inulin and scFOS, we next sought to identify the kinase networks most responsible for distinguishing the kinome profiles between inulin and scFOS. As a proof-of-principle, for each prebiotic, we selected ~20 peptides that were the most differentially phosphorylated compared to the unchallenged or EHEC-challenged controls (10 in the positive and 10 in the negative direction). These kinases were then projected onto Cytoscape to construct protein interaction networks. For both unchallenged and challenged states, the networks between inulin and scFOS differed. The network for scFOS was highly enriched in kinases involved in TLR signaling, including interferon regulatory factor 3 (IRF3), myeloid differentiation primary response gene 88 (Myd88), and receptor for receptor-interacting serine/threonine-protein kinase (RIPK1) (Fig. 2e, g), but the same enrichment was not represented in the networks following inulin exposure (Fig. 2d, f). Moreover, the nodes between inulin- and scFOS-constructed networks also differed in weight distribution, with similarly sized kinase activities in inulin, but more extremes seen in scFOS networks (see IRF3 and IRAK1 in Fig. 2e, g). Thus, not only do inulin and scFOS stratify differently in host GO functional categories and pathways, but they are also characterized by unique protein networks.

### Inulin and scFOS differentially modulate NF-κB, TGFβ, TLR and MAPK pathways

**Fig. 3:**
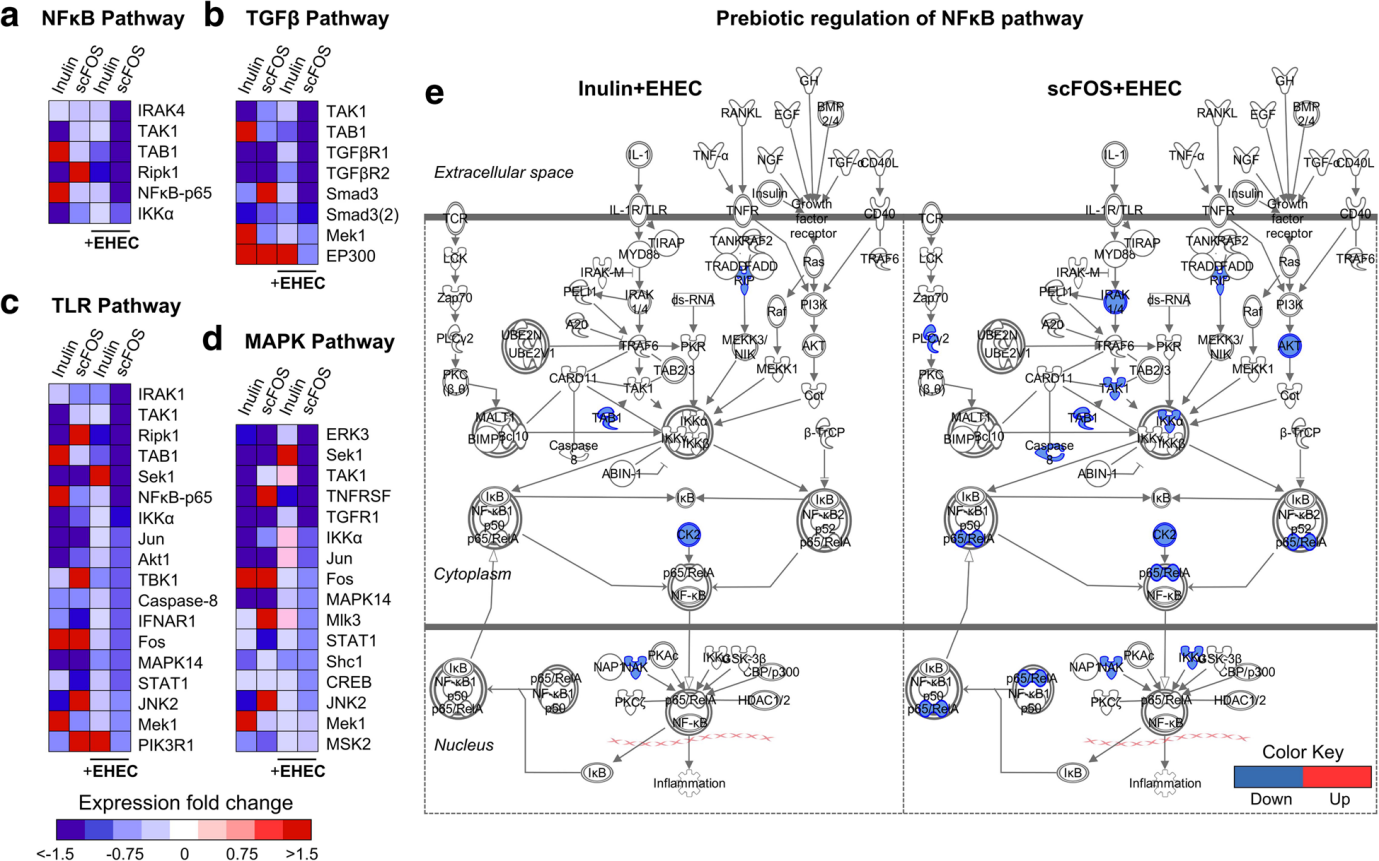
Comparison of the effect inulin and scFOS have on canonical pathways. **a**–**d** Heatmap views illustrating the pathway modulations on NF-κB, TGFβ, TLR and MAPK signaling transductions by inulin and scFOS. **e** Pathway illustration using Ingenuity Pathway Analysis of inulin and scFOS modulation of NF-κB pathway

Given the differences in the functional predictions between inulin and scFOS, next we compared the phosphorylation status of individual peptides within the most highly enriched pathways: MAPK, TGFβ, EGFR and NF-κB. As shown in Fig. 3a, scFOS decreased the phosphorylation of numerous NF-κB signaling mediators including IRAK1, TGFβ-activated kinase (TAK1), TAK-1-binding protein (TAB1), RIPK1, NF-κB-p65 and IκB kinase alpha (IKKα), whereas for inulin-treated cells only RIPK1 and TAB1 were dampened by inulin—suggesting a greater ability for scFOS to inhibit NF-κB signaling. Likewise, this greater response for scFOS can also be seen in TGFβ (Fig. 3b), TLR (Fig. 3c) and MAPK (Fig. 3d) signaling pathways. For example, TLR signaling is involved in the recognition of external stimuli such as EHEC ligands to trigger host innate immune responses [16]. Similar to the trend observed for NF-κB, scFOS decreased the phosphorylation of a greater number of TLR-associated peptides than inulin. These results suggest that scFOS is associated with a more hyporesponsive phenotype by inhibiting host kinase phosphorylation events.

### Prebiotics modulate inflammatory signaling mediators in IECs

**Fig. 4:**
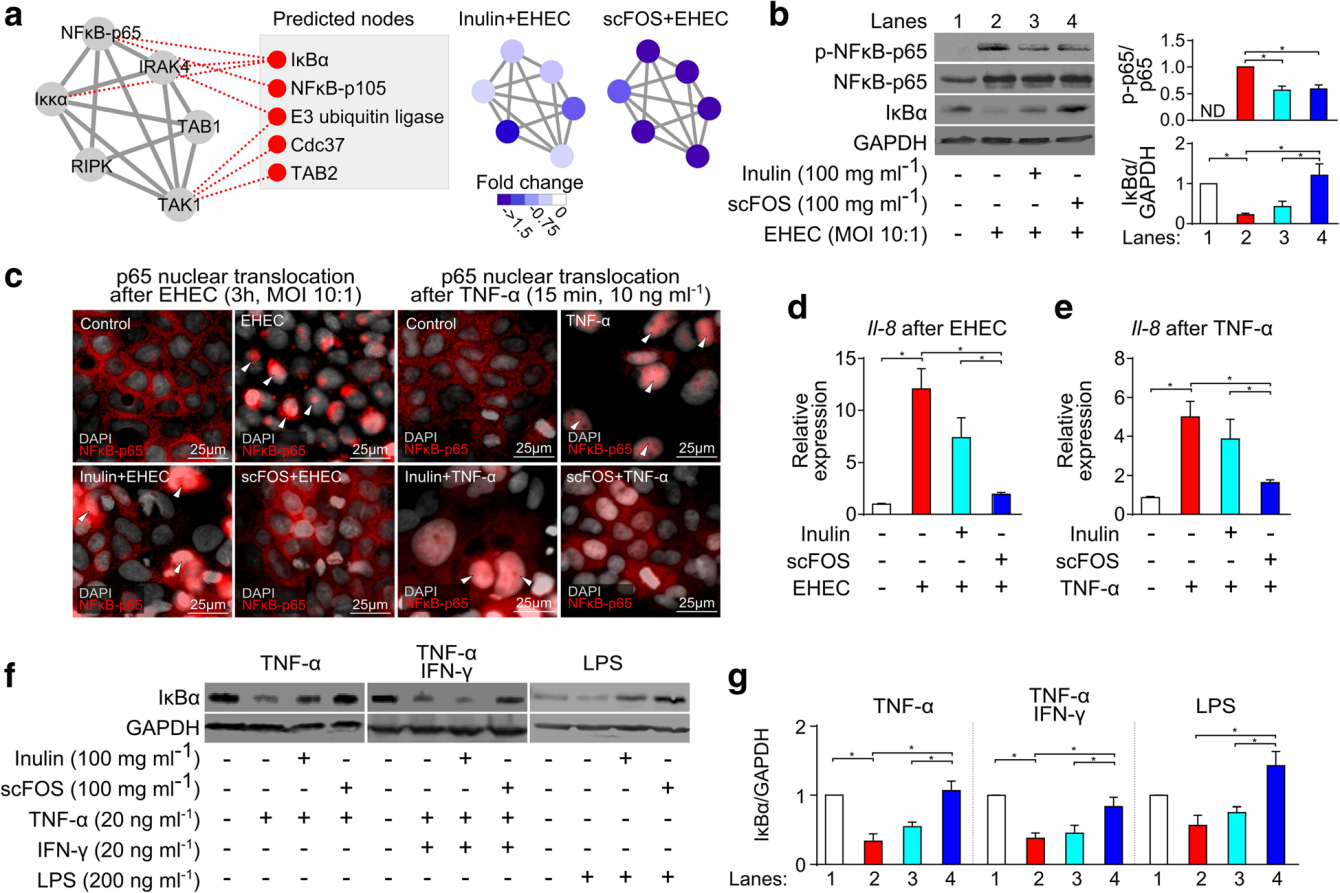
Functional validation of NF-κB pathway in IECs. **a** The NF-κB network with the top five predicted nodes generated from GeneMania. **b** Caco-2Bbe1 cells were treated with inulin or scFOS (10% *w*/*v*, 16 h) before 3 h challenge with EHEC (MOI of 10:1). Cells were then lysed and immunoblotted to check for NF-κB-p65 phosphorylation (*n* = 4) and IKBα degradation (*n* = 5). **c** Localization of NF-κB-p65 in prebiotic-treated Caco-2Bbe1 cells upon 3 h EHEC challenge or 15 min TNF-α challenge (arrows indicate nuclear translocation, representative of 3 separate experiments). **d**, **e** Inulin and scFOS decreased EHEC and TNF-α-triggered *IL-8* expression (*n* = 3). **f**, **g** Caco-2Bbe1 cells pre-exposed to inulin or scFOS (10% *w*/*v*) were challenged with TNF-α and IFN-γ (20 ng ml^−1^ each, 30 min), TNFα alone (20 ng ml^−1^, 15 min) and LPS (200 ng/ml). Cells were lysed and immunoblotted for IKBα levels and GAPDH loading control (*n* = 4). Western blot bands were cropped from the original blots of each individual experiment. Bars represent means ± SEM, *P < 0.05 (ANOVA Bonferonni post hoc test)

To validate the functional predictions from the kinome array, we evaluated NF-κB activation in EHEC-challenged cells using (i) NF-κB western blots, (ii) NF-κB p65 subunit nuclear translocation, and (iii) expression of the downstream NF-κB target gene *IL-8*. To extrapolate from NF-κB predictions, we also constructed the NF-κB protein network using GeneMania [17], and applied a prediction algorithm to generate five new testable targets (Fig. 4a). The target that was most highly predicted was inhibitor of kappa B alpha (IκBα), a known regulator of NF-κB that sequesters the p65 subunit when inactive, but degrades and releases p65 subunit when the NF-κB pathway is activated [18]. As shown in Fig. 4b and 3 h EHEC, EHEC challenge triggered phosphorylation of NF-κB-p65 as well as the degradation of IκBα. As predicted from the kinome results, both inulin and scFOS dampened the phosphorylation of NF-κB-p65, while scFOS but not inulin prevented IKBα degradation. Next, to confirm that scFOS is indeed associated with more hyporesponsive NF-κB signaling, we compared the p65 nuclear translocation and *IL-8* expressions between inulin and scFOS-treated cells. As shown in Fig. 4c, nuclear translocation of p65 was detected in both EHEC control and cells treated with both inulin and EHEC. In contrast, p65 subunit was mainly localized to the cytoplasm within cells treated by scFOS. This difference was recapitulated by qPCR analysis of *IL-8*, whose expression was significantly reduced by scFOS but not by inulin during EHEC challenge (Fig. 4d). Furthermore, we confirmed that this hyporesponsive phenotype is not specific only to EHEC as scFOS also prevented NF-κB p65 nuclear translocation (Fig. 4c) and induction of *IL-8* upon stimulation by tumor necrosis factor alpha (TNF-α) (Fig. 4e), and further limited IκBα degradation when challenged by TNF-α, IFN-γ or LPS (Fig. 4f, g). However, the same effects were again not seen with inulin. Taken together, these findings confirm the kinome data and demonstrate that scFOS evokes a generalized hyporesponsiveness in NF-κB signaling.

In addition to the NF-κB pathway, we also validated the kinome prediction for MAPK signaling (Additional file 2: Figure S2A). P38 MAPKs (otherwise identified as MAPK14 in the kinome result), which transduce extracellular signals from microbial products to activate pro-inflammatory mediators [14, 19], were significantly phosphorylated during EHEC challenge (Additional file 2: Figure S2B, C). However, cells pre-exposed to either inulin or scFOS have decreased phosphorylation of p38 across both the kinome array and western blotting. Similarly, ERK1/2 MAPKs, the most highly predicted target from MAPK interaction network (Additional file 2: Figure S2A), were also dampened by inulin and scFOS (Additional file 2: Figure S2B, C). Therefore, these results demonstrate that prebiotic exposure may directly alter host inflammatory signaling.

### Prebiotics directly dampen LPS-induced inflammation in vivo

**Fig. 5:**
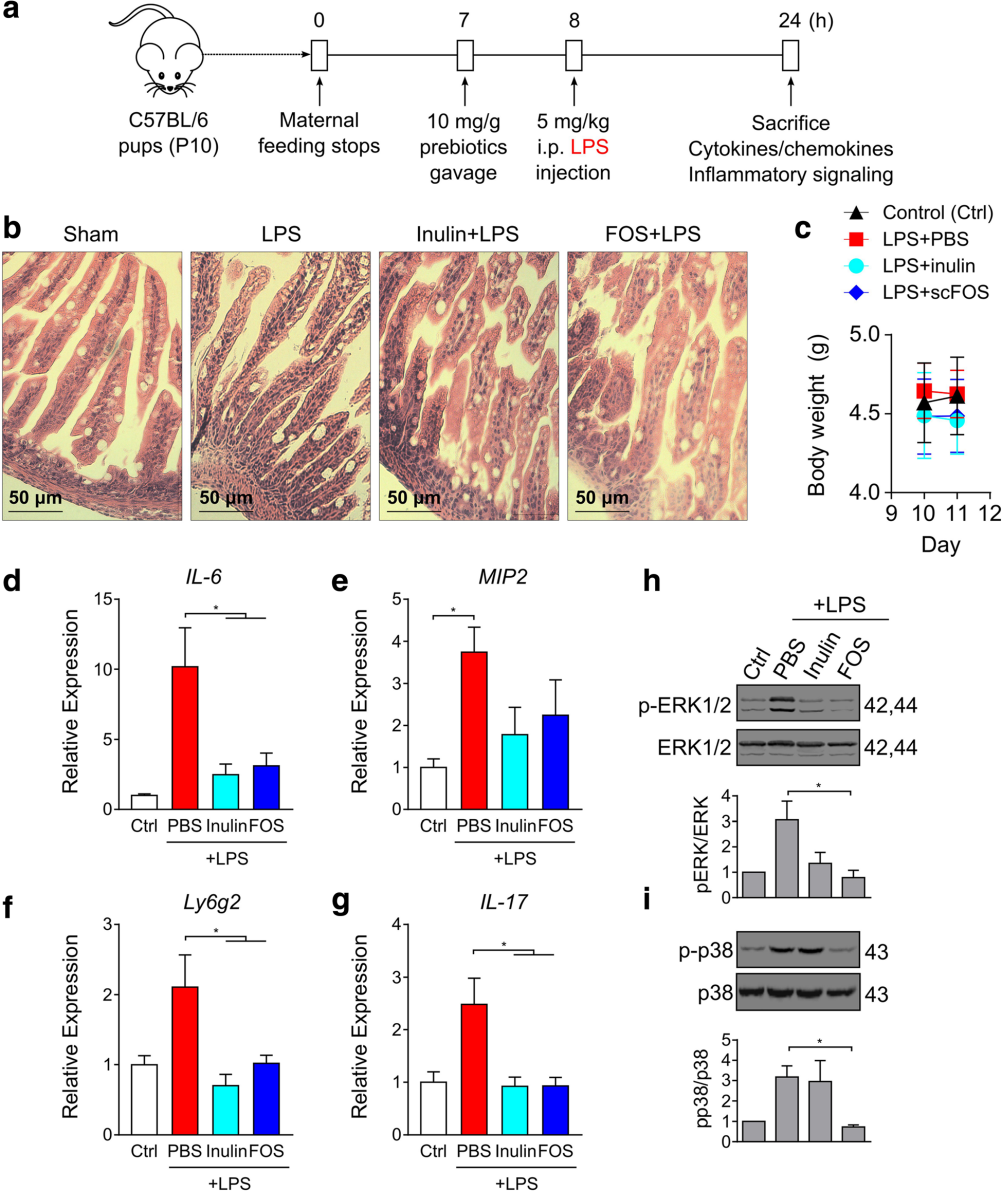
Effect of inulin and scFOS on LPS-induced murine endotoxemia. **a** Diagram illustrating the animal protocol employed to induce murine endotoxemia. **b** Hematoxylin and eosin staining of the terminal ileum from mice gavaged with prebiotics (staining representative of at least five individual animals). **c** Body weights of animals throughout the duration of the study protocol (*n* = 8/group). **d**–**g** RNA extracted from terminal ileum were measured for inflammatory cytokines and chemokines using qRT-PCR (*n* = 8/group). **h**, **i** Terminal ileal sections were lysed and immunoblotted for MAPK phosphorylation (*n* = 4/group). Bars represent means ± SEM, *P < 0.05 (ANOVA Bonferonni post hoc test)

To determine if prebiotic dampening of NF-κB or MAPK can be recapitulated in vivo, we employed a neonatal murine model of endotoxemia whereby pups postnatal day 10 of life were gavaged with a single administration of inulin or scFOS (10 mg/g each) followed by a LPS challenge (Fig. 5a) [13]. Unlike models of colitis, this protocol directly activates host TLR4-NF-κB inflammation in a relatively short response time (~16–24 h)—a timing that is advantageous for the characterization of host inflammation without the expense of significant alterations in the gut microbiota. Given the short LPS exposure, we did not observe any LPS lethality or any changes in ileal histology or body weights following LPS administration (Fig. 5b, c). To assess NF-κB activation, we measured the expression of NF-κB-mediated target genes *IL-6*, macrophage inflammatory protein 2 (*MIP2*, murine analogue of *IL-8*), neutrophil marker *Ly6g2* and *IL-17*. As shown in Fig. 5d–g, inulin and scFOS attenuated LPS-induced *IL-6*, *Ly6g2* and *IL-17* expressions, while *MIP2* showed a downward trend with both prebiotic exposures. To evaluate whether the lower expression of inflammatory cytokines also correlated with the inhibition of MAPKs, we measured the phosphorylation of ERK1/2 and p38 MAPKs in the terminal ileum lysates of the endotoxemic mice. As shown by western blotting (Fig. 5g), LPS injection triggered the phosphorylation of ERK1/2 MAPKs, which were significantly reduced in mice gavaged with both inulin and scFOS. However, the phosphorylation of p38 MAPK was inhibited only by scFOS and not inulin (Fig. 5i). Taken together, these results indicate that prebiotics directly inhibit host NF-κB and MAPK signaling in vivo though the effects on p38 MAPK was specific to scFOS.

### Colonic microbiota relative abundance and function are unchanged by short-term ingestion of inulin or scFOS

**Fig. 6:**
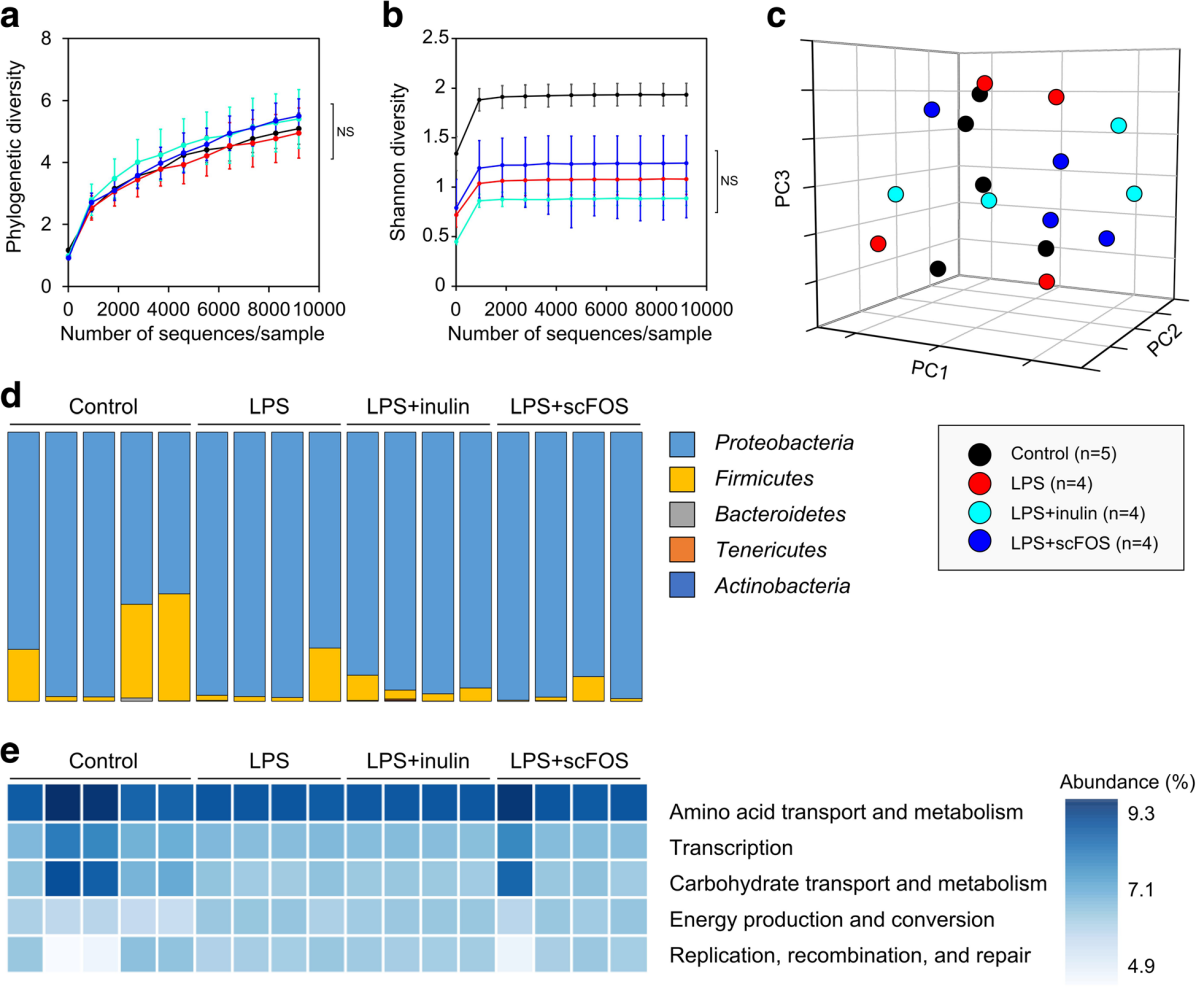
Effects of inulin and scFOS on colonic microbiota of LPS-treated mouse pups. **a** Phylogenetic diversity and **b** Shannon diversity indices for 16S rRNA gene sequences following rarefaction from 10 to 9190 OTU counts/sample (*n* = 4–5/group). Statistical testing was done at a sampling depth of 9194 OTU counts/sample (one-way ANOVA, Bonferroni post hoc test). **c** Unweighted UniFrac principle coordinates analysis of 16S rRNA gene sequences from the colonic microbiota of P10 pups. **d** Relative abundance (%) of phyla in colonic contents of mouse pups at post-natal day 10. **e** Relative abundance (%) of the top five specific COG functional categories for colonic microbiota in P10 pups. Metagenome functional content was predicted from 16S rRNA gene sequences using PICRUSt

Given that long-term feeding of the prebiotics alter the colonic microbiota [1], we reasoned that it is possible that the dampening of host NF-κB and MAPK may be mediated via changes in gut microbiota. To test this possibility, we characterized microbial communities within the colonic segments of endotoxemic mice using 16S rRNA gene sequencing followed by phylogenetic analysis. Comparisons of phylogenetic diversity following rarefaction showed considerable similarity between the four treatment groups (*P* > 0.05, Fig. 6a). Although LPS decreased the Shannon diversity index in colonic microbiota (*P* < 0.01), no detectable differences were seen with inulin and scFOS treatments relative to LPS alone (*P* > 0.05, Fig. 6b). To further compare bacterial diversity between the groups, we performed a cluster analysis using the unweighted UniFrac metric as shown in the principle coordinates analysis (PCoA) plot (Fig. 6c). As shown in the PCoA, colon samples did not appear to cluster according to LPS or prebiotic treatment. This observation was recapitulated at the phylum and genus level, where no significant differences in relative abundance were observed between control and LPS-alone animals as well as between LPS-treated groups with and without prebiotic intake (*Q* > 0.05, Fig. 6d, statistical testing shown in Additional file 3: Figure S3, Additional file 4: Figure S4, Additional file 5: Figure S5). To further characterize the effect of inulin and scFOS on microbial function, we predicted metagenome functional content from 16S rRNA gene sequences using PICRUSt [20]. The top five specific Clusters of Orthologous Groups (COG) functional categories among mouse groups are shown in Fig. 6e. No significant differences in functional content were observed between LPS-treated groups with and without prebiotic intake (*P* > 0.05, Additional file 6: Figure S6 depicts all COG functional categories predicted by the analysis as well as significant differences in functional content between control mice and all LPS groups). Therefore, these results indicate that the intestinal microbiota remained stable and was unaffected by the short-term administration of either inulin or scFOS.

## Discussion

The benefits of prebiotics on host health and disease have traditionally centered on their interactions with resident gut microbes [1]. Emerging evidence suggests prebiotics may also exert direct immune-modulatory effects on the intestinal mucosa, but how these effects are mediated is unclear. To this end, we used a systems biology approach to investigate signaling responses of IECs following exposure to prebiotics. Importantly, we demonstrated that in the absence of microbes, prebiotics directly regulate the phosphorylation of select host signaling molecules to alter mucosal responses to injury. This conclusion is supported by several lines of evidence: firstly, prebiotic-treated IECs exhibited hyporesponsiveness to MAPK and NF-κB activations when challenged by EHEC O157:H7, inflammatory cytokines or LPS. Secondly, inulin and scFOS both altered the phosphorylation status of kinases from several classical pathways of innate immunity. Thirdly, a single oral gavage of prebiotic mitigated LPS-TLR4 inflammation without altering the gut microbiota in newborn pups. Taken together, these results indicate that in addition to manipulating gut microbes, prebiotics also directly impact signaling in the intestinal mucosa.

Prebiotic-mediated immune regulation has been previously described in several disease models, including obesity, diabetes and infectious colitis [21–23]. In a model of obesity and type 2 diabetes, feeding oligofructose to leptin-deficient mice decreased the expression of pro-inflammatory cytokines and markers of oxidative stress [21]. Although these changes were previously linked to the induction of *Bifidobacteria* spp. in the colon, recent studies show that immune changes can also occur independent of the gut microbiota [8, 9, 23, 24], which suggests prebiotics may mediate a direct role on regulating host inflammation. Our study provides a molecular explanation whereby prebiotics directly alter kinome activities to regulate host signaling pathways, including TLRs, MAPKs and NF-κB. This observation agrees with previous in vitro work that have shown monocytes, DCs and IECs can all release cytokines in response to prebiotics and are abolished with TLR4 inhibition using either neutralizing antibodies or siRNAs [5–7, 25].

There is crucial functional relevance of prebiotics downregulating mucosal immune signaling. Mucosal TLR activation contributes to anti-microbial defense and epithelial barrier integrity [16, 26], but excessive activation triggers mucosal inflammation and tissue damage [27]. For example, dysregulated intestinal TLR4 activation generates chronic inflammation and intestinal epithelial apoptosis in the setting of necrotizing enterocolitis (NEC), the current leading cause of death among preterm infants [28]. Interestingly, human milk prebiotic oligosaccharides prevent NEC in rats and piglets [29, 30], possibly via direct immune-modulatory effects which inhibit LPS-induced TLR4 signaling [31]. Clinically, while mixtures of galactooligosaccharides (GOS) and long-chain FOS (9:1) were reported to reduce NEC incidence in very-low-birth-weight infants [32], a similar benefit was not seen with the longer chained inulin [33]. We extended these findings by showing that certain prebiotic structures directly modulate mucosal signaling to facilitate host immune resistance. Dietary prebiotics may thus be crucial immune educators for the neonatal intestine. Further elucidation of the signaling differences between prebiotic structures, particularly in chain lengths, side chains, and human versus plant sources, will be important for designing optimal strategies to prevent inflammatory conditions. Therefore, future studies need to address the structure-function relationship of prebiotics to better delineate underlying mechanism(s) of action.

Interestingly, the most pronounced effect we identified was the downregulation of IRAKs—upstream TLR signal transducers that activate NF-κB and MAPK pathways [34]. In our study, scFOS blunting of IRAK4 phosphorylation paralleled the repression of MAPK and NF-κB, and also correlated with the reduction in *IL-8* expression in Caco-2Bbe1 cells. Similarly, immune reactivity to LPS was attenuated in mouse pups receiving either inulin or scFOS, suggesting a role for prebiotics as TLR regulators to constrain immune-reactivity against external stimuli. Indeed, downregulation of IRAK1 dampens LPS sensitivity in macrophages and IECs, and is critical for immune tolerance [35–37]. On the other hand, uncontrolled activation of select kinases may exacerbate intestinal inflammation. For instance, in the kinome profiles of patients with Crohn’s disease and ulcerative colitis, inflamed tissues have significant activation of p21Rac signaling [38]. Although p21Rac was not one of the targets on our kinome array, several p21Rac downstream targets including p38 MAPK and p21-activated kinase-4 (PAK4) were highly downregulated by inulin and scFOS, suggesting an anti-inflammatory response. This also fits with the previous observations that p38 MAPK inhibitors can significantly reduce inflammation in human endoxtemia [39]. Together, these findings suggest that a tolerogenic effect on host kinome may be mediated by mucosal exposures to prebiotics that are independent of microbiota. However, one caveat to this interpretation is that we did not incorporate germ-free animals in the LPS-endotoxemia challenge. Although we have shown in conventionalized mice that there were no changes in microbial relative abundance or function with inulin and scFOS, we cannot completely exclude other microbial interactions. Validation of these direct kinome-mediated immune responses in germ-free animals could be the subject for future research.

Despite having identical monosaccharide linkages and differing only by lengths, inulin and scFOS induced entirely divergent signaling cascades. ScFOS modulated multiple canonical pathways including MAPK, TLR and TGFβ, whereas inulin affected only the MAPK pathway. Although this difference could be attributed to steric hindrance for receptor binding, it is likely that other factors, such as membrane fluidity or receptor clustering could also be involved [2]. The specific structural requirement for prebiotic effects is further supported by previous studies. For example, only fructans of certain lengths protect barrier function in IECs [25], and only oligosaccharides with sialic acid side-chains reduce the severity of injury in a rat model of NEC [29]. The present work adds another layer of complexity to this structure-function relationship by demonstrating that prebiotic signaling is also highly variable between structures. Whether this variation explains the different clinical utility of therapeutic prebiotic oligosaccharides warrants further research.

Although kinase-phosphorylation events represent an important component of host cell signaling networks, there are 518 identified in humans, each of which harbors multiple phosphorylation sites and identified substrates [11]. The DPPs uncovered here, though relevant, may therefore only be a small sample of the overall kinome changes induced by prebiotics. Additionally, we acknowledge that although kinase-phosphorylation events represent one potential mechanism of prebiotic immunomodulation, there may well be other post-translational modifications involved, such as methylation, ubiquitination and SUMOylation that also regulate host cell signal transductions. For instance, chitosan oligosaccharides (DP = 2–8) were shown to reduce LPS-induced inflammation in vascular endothelial cells by directly suppressing NF-κB-O-GlcNAcylation to dampen inflammatory signaling [40]. Despite these additional possibilities, our study nevertheless provides strong proof-of-principle that prebiotic exposure directly alters the kinome landscape to regulate signaling in the host intestine.

## Conclusions

In conclusion, our findings identify prebiotics as potent modulators of host innate immune pathways to prevent bacterial-, LPS- and cytokine-induced inflammation. Specifically, these changes are due to the direct effects on host kinome activities, which are specific to the structure of the prebiotic. Therefore, these results show that, besides altering the gut microbiota, prebiotics can directly elicit signal transduction effects and facilitate innate immune tolerance to support intestinal homeostasis.

## Methods

### Cell culture

Caco-2Bbe1 human colonic epithelial cells were obtained from American Type Culture Collection (Manassas, VA) and grown at 37 °C in 5% CO_2_ using Dulbecco’s modified Eagle’s medium (DMEM) (Life Technologies, Thermo Scientific, Burlington, Canada) supplemented with 10% fetal bovine serum (Life Technologies), 0.01 mg/ml human transferrin (Sigma, St. Louis, MO), and 1 mM sodium pyruvate (Gibco, Waltham, MA). Cells were seeded onto six-well plates at a density of 5 × 10^5^ cells/well for infection assays and culture media changed every 2 days.

### Prebiotics

As described previously [22], short-chain fructooligosaccharide and inulin were obtained from Nutrition GTC (Golden, Colorado) and Quadra Chimie Lté (Vandreuil, Canada), respectively. ScFOS contains 2–9 degrees of polymerization (DP) of fructose monomers while inulin has a DP of > 10. To treat cells, powdered prebiotics were solubilized in warm (37 °C) culture medium at 10% *w*/*v* and added into wells. For in vivo experiments, prebiotics were solubilized in warm PBS at 10% *w*/*v* and orally gavaged into mouse pups at a concentration of 10 mg/g of body weight.

### Inflammatory challenge

Enterohemorrhagic *Escherichia coli* serotype O157:H7, strain CL56 colonies were grown overnight at 37 °C on Columbia 5% blood agar plates (BBL, Sparks, MD). Prior to the day of infection, individual colonies were grown statically overnight in Penassay broth (Gibco) at 37 °C, and subcultured the next day in Penassay broth for 3 h. As described previously [22], bacterial cells were spun down and pellets resuspended in antibiotic-free DMEM. At a MOI of 10:1, EHEC was added onto Caco-2Bbe1 monolayers for 3 h to monitor IκBα, p-NF-κB, and the phosphorylation of p38 and ERK1/2 MAPKs. To measure *IL-8* expression, EHEC was maintained for 6 h prior to TriZOL extraction (see below). For non-EHEC stimulations of NF-κB, TNF-α (Sigma) was added at 20 ng/ml (at 30 min) to monitor IκBα protein levels, 10 ng/ml (at 15 min) for NF-κB p65 nuclear translocation and 20 ng/ml (at 6 h) to monitor *IL-8* expression; IFN-γ (Sigma) and TNF-α (Sigma) were both added at 20 ng/ml (at 15 min) for IκBα degradation; and LPS (Sigma) was added at 200 ng/ml (at 30 min) to measure IκBα protein degradation.

### Experimental murine endotoxemia

Animal ethics approval for this study was obtained from the Ethics Committee of the Hospital for Sick Children, Toronto, Canada (protocol #32238). All methods performed were carried out in accordance with the approved guidelines and regulations. Protocols were adopted from a previous report [13]. Briefly, wild-type C57BL/6 mice were obtained from Jackson Laboratory (Bar Harbor, ME). Ten-day-old mice were separated from dams and placed nil per os for 6 h. Endotoxemia was induced via an intraperitoneal injection of LPS (5 mg/kg, 16 h, Thermo Scientific). PBS control, inulin or scFOS were orally gavaged 1 h prior to LPS injection. Terminal ileal sections were stored at − 80 °C and used for histological examination, cytokine analysis via qPCR and western blotting to determine MAPK activation. Protein lysis of mouse terminal ileum was done by suspending 5 mm of ileal segments in RIPA-lysis buffer with 30 s of sonication.

### Immunoblotting

Caco-2Bbe1 cells were lysed using 2% NP-40 RIPA buffer added with protease and phosphatase inhibitors, as described elsewhere [41]. Lysates were separated using sodium dodecyl sulfate polyacrylamide gel electrophoresis (SDS-PAGE) and separated protein contents transferred onto nitrocellulose membranes (BioTrace NT). Primary antibodies used include mouse anti-p-NF-κB-p65 (Cell Signaling, Danvers, MA), rabbit anti-IκBα (Cell Signaling), rabbit anti-p-ERK 1/2 (Cell Signaling), rabbit anti-ERK 1/2 (Cell Signaling), rabbit anti-p-p38 (Cell Signaling), rabbit anti-p38 (Cell Signaling), mouse anti-GAPDH (Santa Cruz, Dallas, TX) and anti-NF-κB-p65 (Santa Cruz). Membranes were washed the next day and visualized using IRDye 680 goat anti-rabbit immunoglobulin G (IgG) (1:10,000 dilution) and IRDye 800 goat anti-mouse IgG secondary antibodies (1:10,000 dilution) (Rockland Immunochemicals, Gilbertsville, PA). Blots were analyzed using an Odyssey imaging system (LI-COR Biosciences, Lincoln, NE). Band intensities were measured using *Gels Analyze* tool in ImageJ version 1.48v (NIH), and densitometry ratios were derived by dividing band intensities to respective loading controls and expressed as fold change to control lanes (set at 1).

### Immunofluorescence

As previously described [22], Caco-2Bbe1 cells were grown using 1-mm glass coverslips and fixed for 10 min at 4 °C using paraformaldehyde. To immunostain for NF-κB-p65 nuclear translocation, cell monolayers were blocked using 3% bovine serum albumin (BSA, Sigma) and incubated with rabbit anti-NF-κB-p65 antibody (Santa Cruz) for 1 h. Cells were washed with cold PBS and incubated with Alexa fluor 488-conjugated goat anti-rabbit secondary antibody (1:5000) (Life Technologies). Cells were washed with cold PBS and added with 4′,6-diamidino-2-phenylindole (DAPI, Life Technologies) and mounted onto glass slides with ProLong antifade mounting solution (Molecular Probes®, Life Technologies). Images were taken using a Leica DMI6000B fluorescence microscope and companion DFC 360FX camera (Leica Microsystems, Concord, Canada).

### qRT-PCR

RNA extraction for both Caco-2Bbe1 cells and murine ileal segments was performed using TriZOL per manufacture’s protocol (Thermo Fischer). All extracted RNA samples were standardized to 50 ng/μl and reverse-transcribed to cDNA samples using iScript cDNA synthesis system (Bio-Rad, Hercules, CA) as described previously [22]. qRT-PCR reactions were performed on a CFX96 C1000 Thermal Cycler (Bio-Rad) using Ssofast Evagreen Supermix (Bio-Rad). The following primers (5′-3′) were used:

*human-GAPDH*, ACCCACTCCTCCACCTTTGAC (forward), CCACCACCCTGTTGCTGTAG (reverse); *human-β-actin*, CTGGAACGGTGAAGGTGACA (forward), AAGGGACTTCCTGTAACAATGCA (reverse); *human*-*IL-8*, ACTGAGAGTGATTGAGAGTGGAC (forward); AACCCTCTGCACCCAGTTTTC (reverse);

*Murine-GAPDH,* TGAAGCAGGCATCTGAGGG (forward), CGAAGGTGGAAGAGTGGGAG (reverse); *Murine-IL6,* CCAATTTCCAATGCTCTCCT (forward), ACCACAGTGAGGAATGTCCA (reverse); *Murine-MIP2*, *AAAATCATCCAAAAGATACTGA* (forward), CTTTGGTTCTTCCGTTGAGG (reverse); *Murine-IL-17,* CTTGGCGCAAAAGTGA (forward); TTGCTGGATGAGAACAGAA(reverse); *Murine-Ly6G2*, *TGCGTTGCTCTGGAGATAGA* (forward); *CAGAGTAGTGGGGCAGATGG* (reverse).

Expression levels were calculated by the ∆∆C_t_ method and normalized to reference housekeeping genes (human: *GAPDH* and *β-actin*, mouse: *GAPDH*).

### Kinome array

Methods for the human peptide array were based on the protocol described previously [12, 42]. Briefly, approximately 10^6^ Caco-2Bbe1 cells were seeded per well into six-well-tissue culture plates. Upon reaching confluence, cells were exposed to inulin or scFOS (10% *w*/*v*, 16 h) prior to 3 h challenge with EHEC (MOI of 10:1). Cells were then lysed using lysis buffer containing 20 mM Tris-HCl [pH 7.5], 150 mM NaCl, 1 mM EDTA, 1% Triton, 2.5 mM sodium pyrophosphate, 1 mM Na_3_VO_4_, 1 mM NaF, 1 μg/ml leupeptin, 1 g/ml aprotinin and 1 mM PMSF (all from Sigma). Protein samples from three separate experiments were spotted onto the peptide array for 2 h at 37 °C, and a total of 18 arrays (3 × biological replicates, 6 × biological conditions) were applied with a phospho-specific fluorescent ProQ Diamond phosphoprotein stain (Invitrogen). Arrays were washed three times with 20% acetonitrile before being dried completely. Phosphorylation levels were read using a GenePix Professional 4200A microarray scanner (MDS Analytical Technologies). Intensity signals were outputted as mean pixel intensity after subtraction of background subtraction with default scanner saturation levels, and averages of the nine intra-array technical replicates were used. Statistical testing, hierarchical clustering and coordinates for PCoA analysis were further processed through PIIKA2 (http://saphire.usask.ca/saphire/piika/), a web-based pipeline for kinome array processing [12].

### Kinome data analyses

To identify peptides with significant changes, a paired *t-*test was performed through PIIKA2 to compare phosphorylation intensities of prebiotic conditions to non-prebiotic control conditions. The specific comparisons were inulin versus control, scFOS versus control, inulin plus EHEC versus EHEC alone, and scFOS plus EHEC versus EHEC alone. A significance threshold level of 0.010 was chosen as previously described to minimize false negatives and retain data coverage for subsequent pathway analyses [43, 44]. The identified peptides had high technical reproducibility (average of 260.7 out of 282 peptides were consistently phosphorylated over the nine intra-array technical replicates according to a *χ* ^2^ test) and were further analyzed for gene ontology and pathway annotations using DAVID [15]. For data visualization in heatmap views, peptide phosphorylation intensities, fold changes and −log *P* values were uploaded onto GenePattern to generate heatmaps using the tool *Interactive Heatmap Viewer* [45]. Visualization for the NF-κB pathway was performed using a commercial copy of Ingenuity Pathway Analysis (Qiagen Bioinformatics, Redwood City, CA). Protein interaction networks were constructed using the web-based gene network prediction tool GeneMania (http://genemania.org/) [17]. DPPs were uploaded in the form of UniProt IDs with specific parameters set at *Pathway* for protein interactions, and a maximum of five for *max resultant genes* predicted using *automatic weighting* [17]. Networks generated were uploaded and visualized using Cytoscape version 3.0 [46].

### Microbiota profiling

Whole colon segments from P10 mice were excised at sacrifice and immediately snap frozen and stored at − 80 °C. Genomic DNA was extracted from mouse colon samples as described in Whelan et al. but with 2.8-mm ceramic beads in addition to 0.1-mm glass beads and processing using the MagMAX Express-96 Deep Well Magnetic Particle Processor from Applied Biosystems with the DNA Multi-Sample kit (Life Technologies #4413022) [47]. 16S rRNA gene amplification and sequencing, read preprocessing, QIIME operational taxonomic unit (OTU) picking, and downstream alpha and beta diversity analyses were carried out for colonic segments as previously described but with amplicon normalization using the SequalPrep Normalization Plate Kit (ThermoFisher #A1051001), the 2013 Greengenes reference database, and a sampling depth of 9194 OTU counts/sample [47]. Statistical testing of alpha diversity metrics was performed using GraphPad Prism 6 (GraphPad Software, La Jolla, CA). The cluster analysis was performed using the unweighted UniFrac metric. PCoA coordinates from this analysis were replotted using OriginPro 8.0 (Origin Labs, Northampton, MA). Metagenome functional content was predicted from 16S rRNA gene sequencing data and reference genomic content using PICRUSt and the clusters of orthologous groups (COG) database (quality statistics shown in Table 1) [20, 48]. Differences in relative abundance of taxa and COG functional categories were calculated and visualized using STAMP [49].

**Table 1:** Quality statistics for metagenome functional content prediction from 16S rRNA gene sequences using PICRUSt. NSTI (Nearest Sequenced Taxon Index) values indicate the average branch length that connects each OTU to the closest reference bacterial genome available during functional content prediction

Treatment group	Mean	Standard deviation	*n*
Control	0.067	0.008	5
LPS	0.093	0.007	4
LPS + inulin	0.096	0.003	4
LPS + scFOS	0.088	0.016	4

### Statistical analysis

Results were expressed as means ± SEM and were derived from at least three separate experiments. All comparisons were made using GraphPad Prism version 6 and STAMP for 16S rRNA gene sequencing data [49]. Comparisons of multiple groups were done using one-way ANOVA with Bonferonni post hoc testing (*P* < 0.05 deemed as statistically significant). Comparisons of two groups were done using Welch’s two-sided *t*-tests with Welch’s inverted 95% CI and Benjamin-Hochberg FDR correction (*Q* < 0.05 significance threshold). For kinome array results, a *P* value threshold of 0.10 was applied to optimize pathway coverages as previously described [43, 44].

## Additional files

Additional file 1: Figure S1. Kinome differences between inulin and scFOS. (A) Phosphorylation intensities of the DPPs identified were converted to fold changes to untreated or EHEC-challenged controls and plotted into a heatmap (*n* = 3, *P* < 0.1, t-test). (B-E) Volcano plots displaying (in red or blue) the DPPs identified in (A) with the top 3 DPPs listed below. (Inu denotes inulin; scF denotes scFOS). (JPEG 1036 kb)

Additional file 2: Figure S2. Functional validation of MAPK pathway in IECs. (A) The MAPK network with the top five predicted nodes generated from GeneMania. (B-C) Inulin and scFOS both decreased EHEC-induced phosphorylation of MAPK14 (p38) and ERK1/2 MAPKs (*n* = 4). Western blot bands were cropped from original blots of each individual experiment. Bars represent means ± SEM, * *P* < 0.05 (ANOVA Bonferonni post hoc test). (JPEG 1086 kb)

Additional file 3: Figure S3. Effects of LPS-induced murine endotoxemia on colonic microbiota. Comparison of the mean relative abundance (%) of (A) phyla and (B) genera (top ten by effect size were shown) between the colonic contents of mouse pups with (LPS) and without (Control) LPS-induced endotoxemia (*n* = 4-5/group, Welch’s two-sided t-test, Welch’s inverted 95% CI, Benjamini-Hochberg FDR correction). (JPEG 162 kb)

Additional file 4: Figure S4. Effects of inulin on colonic microbiota of LPS-treated mouse pups. Comparison of the mean relative abundance (%) of (A) phyla and (B) genera (top ten by effect size were shown) between the colonic contents of mouse pups with (LPS + inulin) and without (LPS) inulin intake before LPS-induced endotoxemia (*n* = 4-5/group, Welch’s two-sided t-test, Welch’s inverted 95% CI, Benjamini-Hochberg FDR correction). (JPEG 165 kb)

Additional file 5: Figure S5. Effects of scFOS on colonic microbiota of LPS-treated mouse pups. Comparison of the mean relative abundance (%) of (A) phyla and (B) genera (top ten by effect size were shown) between the colonic contents of mouse pups with (LPS + scFOS) and without (LPS) scFOS intake before LPS-induced endotoxemia (*n* = 4-5/group, Welch’s two-sided t-test, Welch’s inverted 95% CI, Benjamini-Hochberg FDR correction). (JPEG 157 kb)

Additional file 6: Figure S6. Effects of inulin and scFOS on metagenome functional content of LPS-treated mouse pups. (A) Relative abundance (%) of COG functional categories in colonic contents of P10 pups. Metagenome functional content was predicted from 16S rRNA gene sequences using PICRUSt. Stcvhatistical testing was done using one-way ANOVAs. (B) Comparison of the mean relative abundance (%) of COG functional categories between colonic microbiota of mouse pups with (All LPS groups) and without (Control) LPS-induced endotoxemia (*n* = 4-5/group, Welch’s two-sided t-test, Welch’s inverted 95% CI, Benjamini-Hochberg FDR correction). (JPEG 236 kb)
