# Supplementary figures and images for: Non-digestible oligosaccharides directly regulate host kinome to modulate host inflammatory responses without alterations in the gut microbiota
Source: Microbiome. 2017 Oct 10;5:135. doi: 10.1186/s40168-017-0357-4 (PMC5635512; doi:10.1186/s40168-017-0357-4)

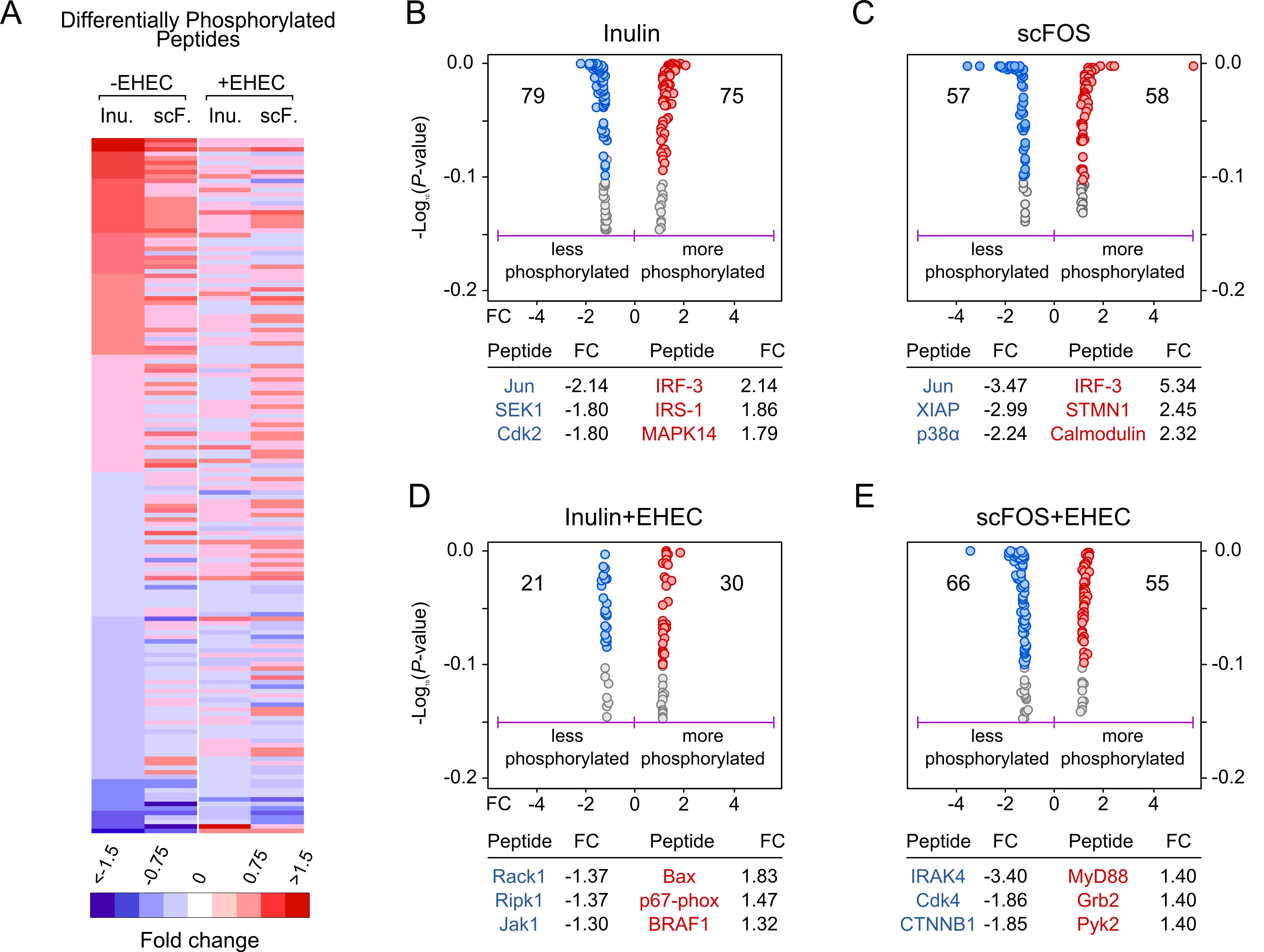

Supplement: Supplementary file 1 — Kinome differences between inulin and scFOS. (A) Phosphorylation intensities of the DPPs identified were converted to fold changes to untreated or EHEC-challenged controls and plotted into a heatmap (n = 3, P < 0.1, t-test). (B-E) Volcano plots displaying (in red or blue) the DPPs identified in (A) with the top 3 DPPs listed below. (Inu denotes inulin; scF denotes scFOS). (JPEG 1036 kb) [file 40168_2017_357_MOESM1_ESM.jpg]

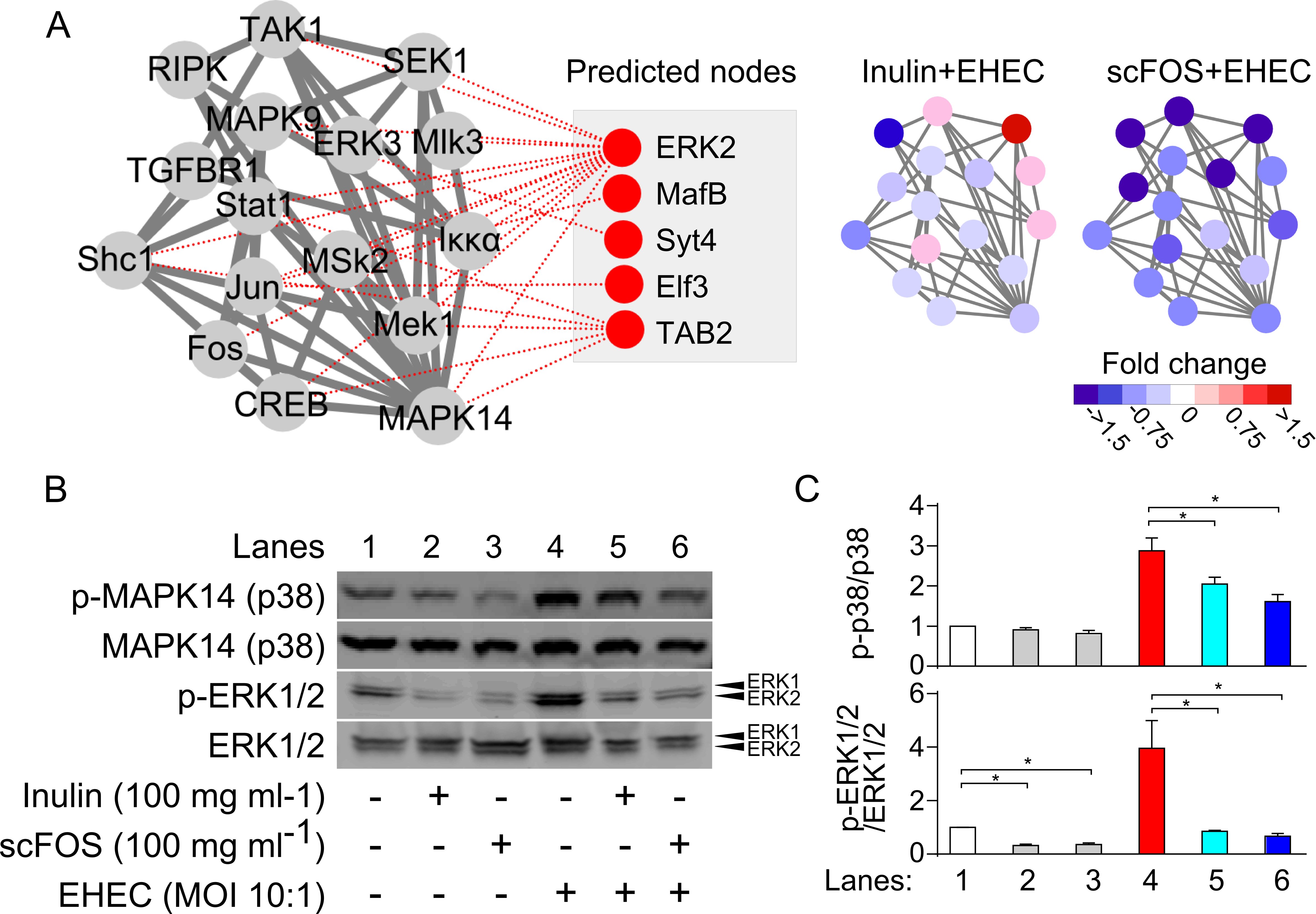

Supplement: Supplementary file 2 — Functional validation of MAPK pathway in IECs. (A) The MAPK network with the top five predicted nodes generated from GeneMania. (B-C) Inulin and scFOS both decreased EHEC-induced phosphorylation of MAPK14 (p38) and ERK1/2 MAPKs (n = 4). Western blot bands were cropped from original blots of each individual experiment. Bars represent means ± SEM, * P < 0.05 (ANOVA Bonferonni post hoc test). (JPEG 1086 kb) [file 40168_2017_357_MOESM2_ESM.jpg]

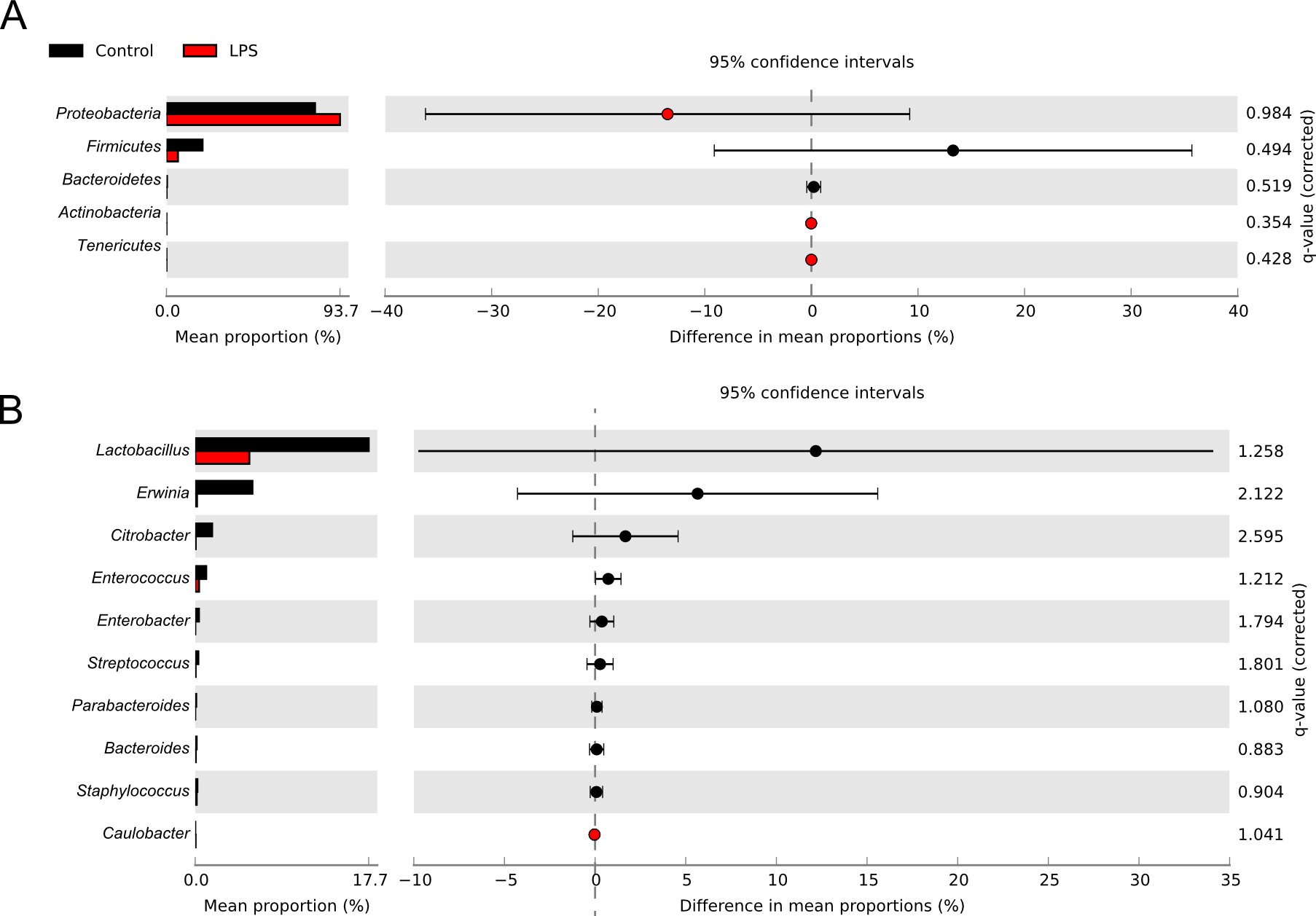

Supplement: Supplementary file 3 — Effects of LPS-induced murine endotoxemia on colonic microbiota. Comparison of the mean relative abundance (%) of (A) phyla and (B) genera (top ten by effect size were shown) between the colonic contents of mouse pups with (LPS) and without (Control) LPS-induced endotoxemia (n = 4-5/group, Welch’s two-sided t-test, Welch’s inverted 95% CI, Benjamini-Hochberg FDR correction). (JPEG 162 kb) [file 40168_2017_357_MOESM3_ESM.jpg]

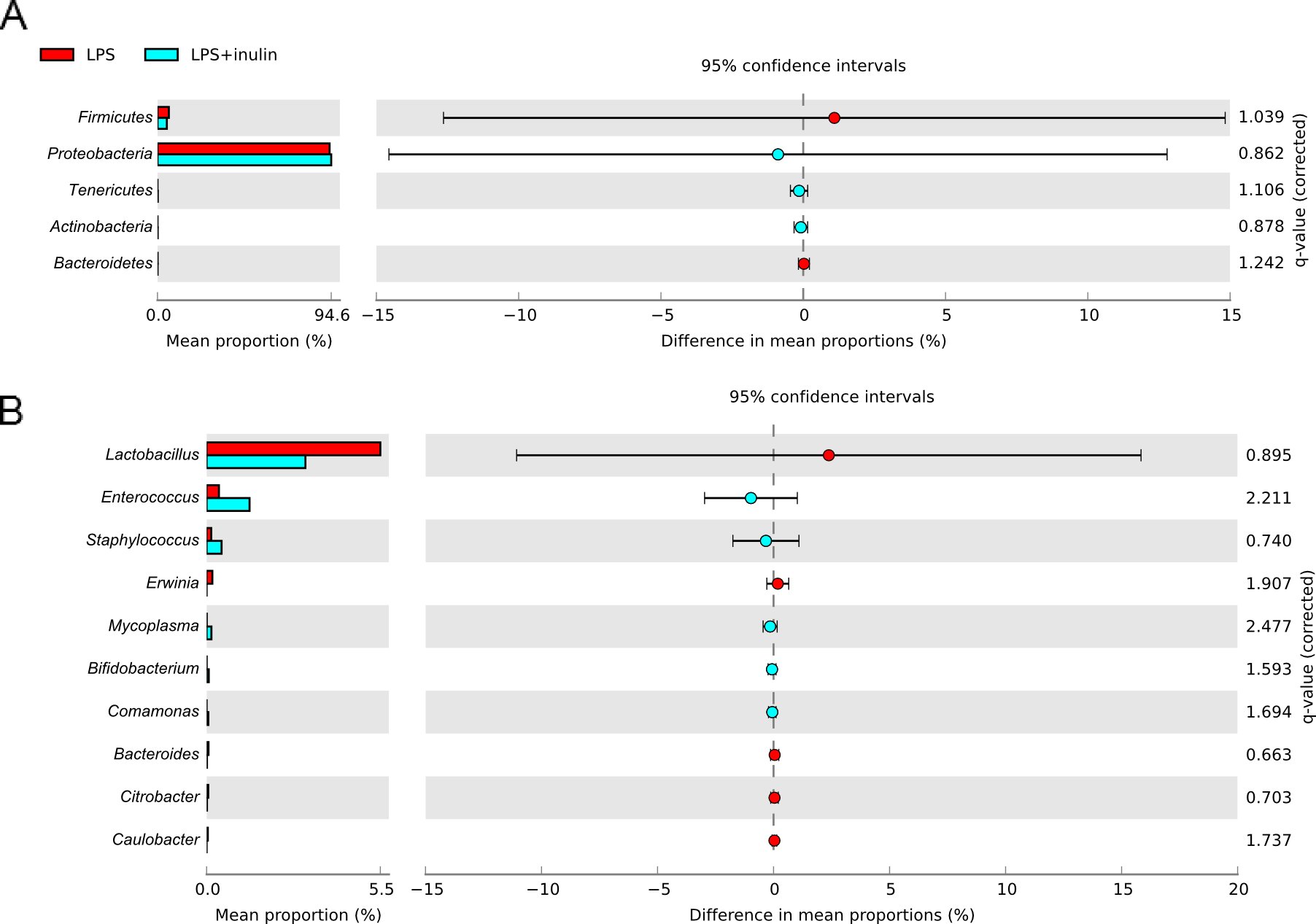

Supplement: Supplementary file 4 — Effects of inulin on colonic microbiota of LPS-treated mouse pups. Comparison of the mean relative abundance (%) of (A) phyla and (B) genera (top ten by effect size were shown) between the colonic contents of mouse pups with (LPS + inulin) and without (LPS) inulin intake before LPS-induced endotoxemia (n = 4-5/group, Welch’s two-sided t-test, Welch’s inverted 95% CI, Benjamini-Hochberg FDR correction). (JPEG 165 kb) [file 40168_2017_357_MOESM4_ESM.jpg]

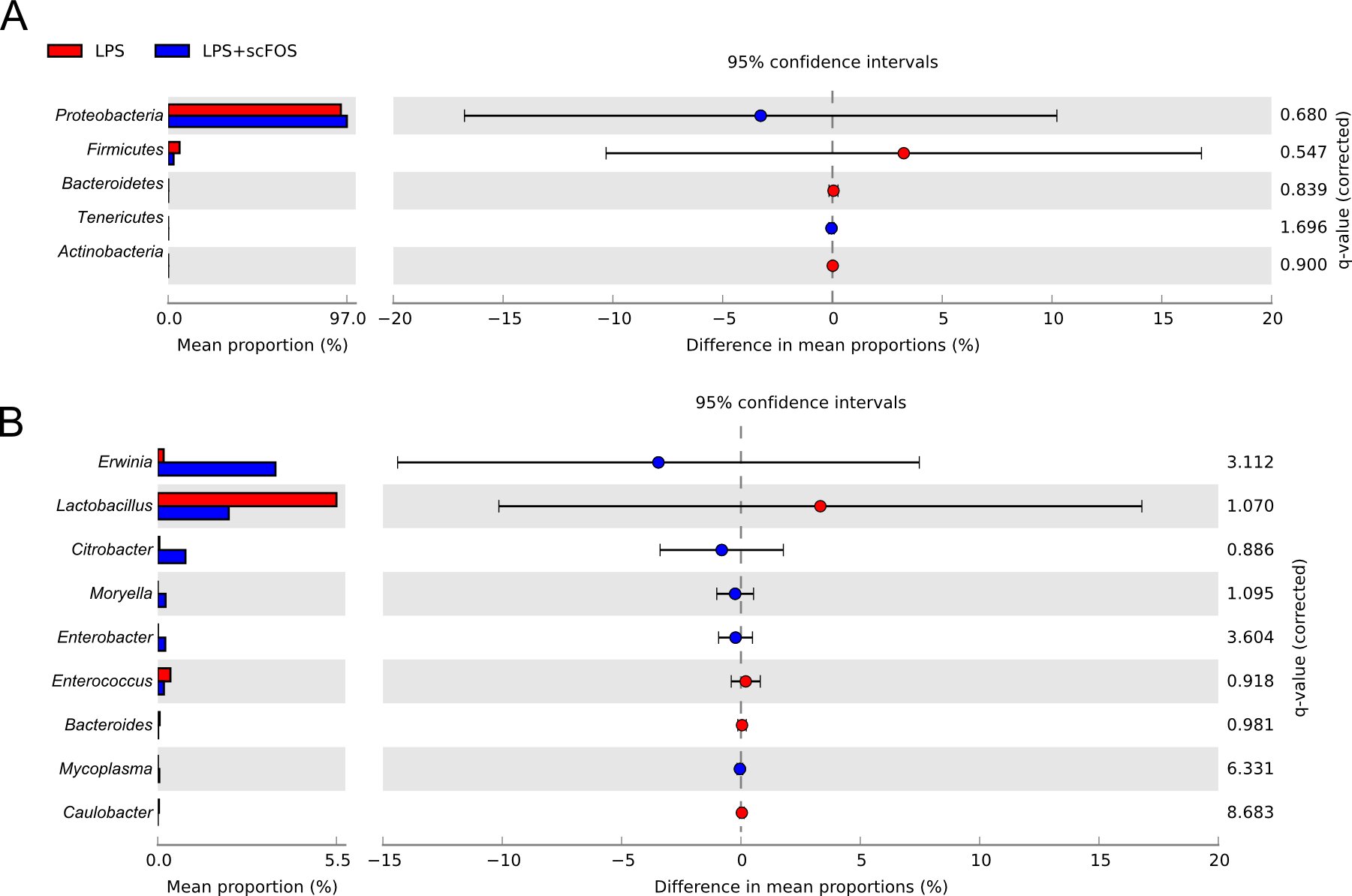

Supplement: Supplementary file 5 — Effects of scFOS on colonic microbiota of LPS-treated mouse pups. Comparison of the mean relative abundance (%) of (A) phyla and (B) genera (top ten by effect size were shown) between the colonic contents of mouse pups with (LPS + scFOS) and without (LPS) scFOS intake before LPS-induced endotoxemia (n = 4-5/group, Welch’s two-sided t-test, Welch’s inverted 95% CI, Benjamini-Hochberg FDR correction). (JPEG 157 kb) [file 40168_2017_357_MOESM5_ESM.jpg]

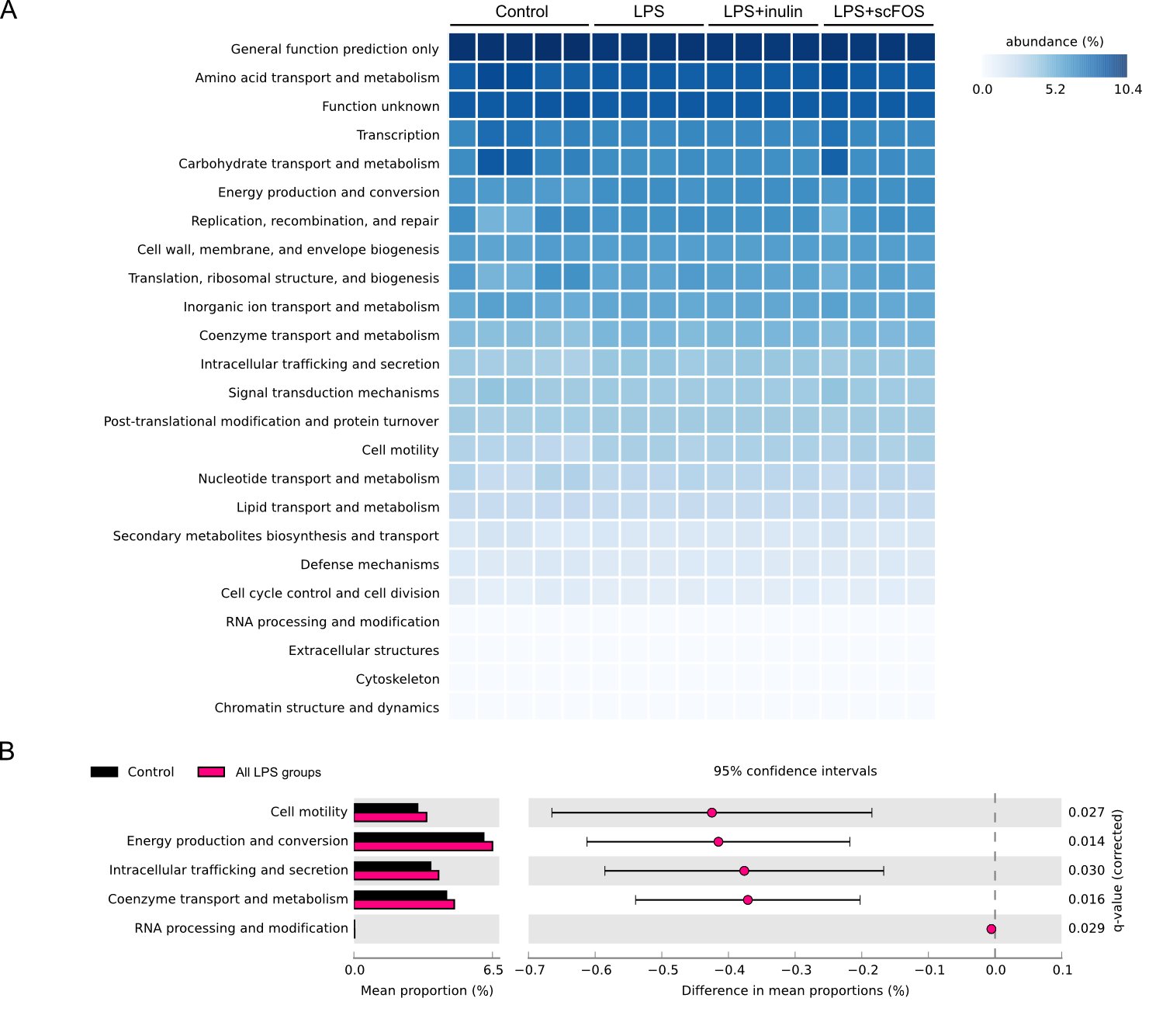

Supplement: Supplementary file 6 — Effects of inulin and scFOS on metagenome functional content of LPS-treated mouse pups. (A) Relative abundance (%) of COG functional categories in colonic contents of P10 pups. Metagenome functional content was predicted from 16S rRNA gene sequences using PICRUSt. Stcvhatistical testing was done using one-way ANOVAs. (B) Comparison of the mean relative abundance (%) of COG functional categories between colonic microbiota of mouse pups with (All LPS groups) and without (Control) LPS-induced endotoxemia (n = 4-5/group, Welch’s two-sided t-test, Welch’s inverted 95% CI, Benjamini-Hochberg FDR correction). (JPEG 236 kb) [file 40168_2017_357_MOESM6_ESM.jpg]
